# A Rate-Adaptive MAC Protocol for Flexible OFDM-PONs

**DOI:** 10.3390/s26010133

**Published:** 2025-12-24

**Authors:** Zhe Zheng, Yingying Chi, Xin Wang, Junjie Zhang

**Affiliations:** 1 Beijing Smartchip Microelectronics Technology Company Ltd., Beijing 100192, China; zhengzhe@sgchip.sgcc.com.cn (Z.Z.); chiyingying@sgchip.sgcc.com.cn (Y.C.); 2 Tianjin Center of National Digital Switching System Engineering and Technological R&D Center, Information Technology Innovation Center of Tianjin Binhai New Area, Tianjin 300457, China; xin.wang@sdichips.com; 3 Key Laboratory of Specialty Fiber Optics and Optical Access Networks, Joint International Research Laboratory of Specialty Fiber Optics and Advanced Communication, Shanghai University, Shanghai 200444, China

**Keywords:** rate-adaptive, MAC protocol, OFDM-PON

## Abstract

**Highlights:**

**What are the main findings?**

- A novel TDM-based, rate-adaptive MAC protocol for OFDM-PONs was designed, featuring a new physical adaptation sublayer and a discretized rate-stage model to manage variable transmission speeds.
- The OLT-side protocol was fully implemented in FPGA and experimentally validated, demonstrating flexible net data rate switching on the downlink from 8.1 Gbit/s to 32.8 Gbit/s.

**What are the implications of the main findings?**

- This work provides a complete, hardware-verified solution that bridges the critical gap between flexible OFDM physical layer advancements and practical, system-level network deployment.
- The protocol enables next-generation optical access networks to efficiently manage multiple users at diverse, distance-dependent data rates, which is a foundational requirement for deploying OFDM-PONs.

**Abstract:**

The practical deployment of Orthogonal Frequency Division Multiplexing Passive Optical Networks (OFDM-PONs) is hindered by the lack of a Medium Access Network (MAC) protocol capable of managing their flexible, distance-dependent data rates, despite their high spectral efficiency. This paper proposes and validates a novel rate-adaptive, Time Division Multiplexing (TDM)-based MAC protocol for OFDM-PON systems. A key contribution is the design of a three-layer header frame structure that supports multi-ONU data scheduling with heterogeneous rate profiles. Furthermore, the protocol incorporates a unique channel probing mechanism to dynamically determine the optimal transmission rate for each Optical Network Unit (ONU) during activation. The proposed Optical Line Terminal (OLT) side MAC protocol has been fully implemented in hardware on a Xilinx VCU118 FPGA platform, featuring a custom-designed ring buffer pool for efficient multi-ONU data management. Experimental results demonstrate robust upstream and downstream data transmission and confirm the system’s ability to achieve flexible net data rate switching on the downlink from 8.1 Gbit/s to 32.8 Gbit/s, contingent on the assigned rate stage.

## 1. Introduction

The exponential growth of internet traffic, driven by emerging high-bandwidth applications such as 4 K/8 K video streaming, virtual and augmented reality, and multimedia conferencing, has placed unprecedented demands on network infrastructure [1]. The access network, often termed the “last mile,” is a critical bottleneck in this ecosystem. While Passive Optical Networks (PONs) have become the primary solution for Fiber-to-the-Home deployments due to their cost-effectiveness and reliability, conventional technologies are reaching their limits [2,3]. Time Division Multiplexing PONs (TDM-PONs) face increasing complexity in burst-mode reception and scheduling at higher speeds, while Wavelength Division Multiplexing PONs (WDM-PONs) lack flexible, fine-grained bandwidth allocation capabilities [4].

Orthogonal Frequency Division Multiplexing PON (OFDM-PON) has emerged as a compelling candidate for the next generation of optical access networks [5,6]. By partitioning a broadband channel into numerous orthogonal subcarriers, OFDM-PON offers significant advantages, including high spectral efficiency, excellent resilience to chromatic dispersion, and exceptionally flexible resource allocation. The ability to dynamically adjust modulation formats, power, and the number of bits per subcarrier allows the network to adapt to varying channel conditions and service requirements, optimizing performance for diverse users and distances.

Despite extensive research demonstrating high-capacity physical layer transmissions in OFDM-PON systems, with speeds exceeding 50 Gbps per wavelength [7], a critical gap remains at the data link layer. Most existing studies focus on PHY performance optimization [8,9,10,11,12], neglecting the development of a corresponding Medium Access Control (MAC) protocol required for practical system operation [13,14,15]. To ensure compatibility with existing PON interoperability protocols, the MAC frame structure based on XG-PON was proposed for [16,17] for upstream and downstream direction. However, the existing papers rarely focus on the specific implementation schemes for the registration and access control procedure for the OFDM-PON system. The inherent flexibility of the OFDM-PON physical layer, which results in variable and distance-dependent data rates, introduces a significant challenge in heterogeneous rate management at the MAC layer. This challenge is absent in fixed-rate TDM-PON systems, rendering their MAC protocols fundamentally inapplicable.

This paper addresses this gap by designing, implementing and validating a complete, rate-adaptive MAC protocol tailored for flexible OFDM-PON systems. Our key contributions are:

1. A novel TDM-based MAC protocol architecture featuring a new physical adaptation sublayer to manage variable data rates.
2. A flexible, three-layer frame header structure that efficiently communicates rate-stage and scheduling information for multiple Optical Network Units (ONUs).
3. A unique channel probing mechanism embedded within the ONU activation phase to facilitate rate-adaptive link initialization, overcoming the limitations of fixed-rate PONs by dynamically ascertaining the optimal transmission profile for each ONU.
4. A full-scale hardware realization of the Optical Line Terminal (OLT) side protocol on an FPGA platform, substantiating the practical feasibility of complex rate-adaptive scheduling logic and bridging the gap between theoretical models and system-level deployment.

## 2. Protocol Design and Architecture

The proposed MAC protocol is designed to bridge the functional gap between the upper network layers and the flexible-rate OFDM-PON physical layer. It builds upon the foundational principles of GPON while introducing novel elements to manage rate diversity.

### 2.1. Protocol Stack and Rate-Stage Model

**Figure 1:**
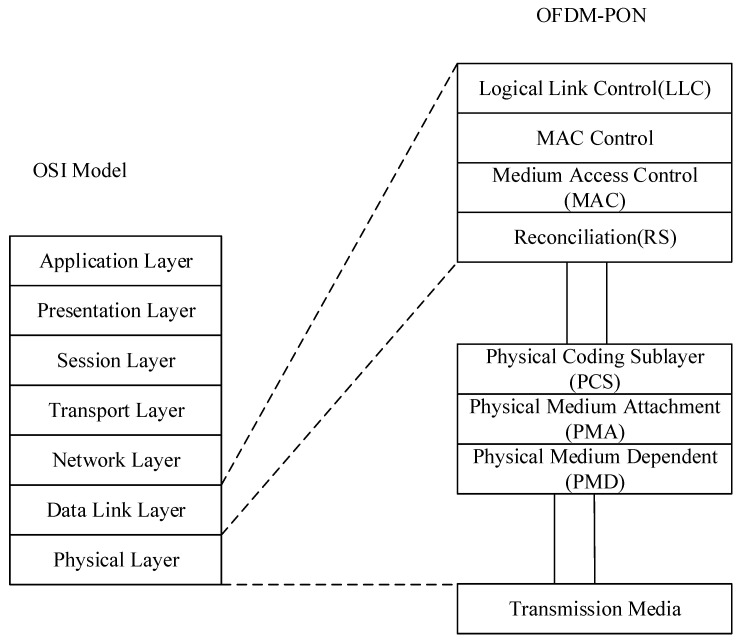
OFDM-PON Protocol Stack.

The protocol stack is situated within the physical and data link layers of the OSI model, as shown in Figure 1. It is composed of an adaptation sublayer, a framing sublayer, and a crucial physical adaptation sublayer. The adaptation sublayer utilizes the GPON Encapsulation Method (GEM) to uniformly package variable-length Ethernet frames from higher layers.

In the physical layer portion of the OSI reference model, the protocol architecture of OFDM-PON comprises the following sublayers and interfaces from the lower to the higher levels: the Medium Dependent Interface (MDI), the Physical Medium Dependent sublayer (PMD), the Physical Medium Attachment sublayer (PMA), and the Physical Coding Sublayer (PCS). The MDI defines the characteristics of signals transmitted over the physical medium and the electrical interface between the medium and devices and is primarily responsible for the adaptation between the signal and the physical medium. The PMD sublayer handles the adaptation between the physical layer and the transmission medium. The PMA sublayer is responsible for signal transmission and reception, timing recovery, and phase alignment, managing the transfer of physical signals. The PCS sublayer converts data bits into coding formats suitable for transmission over the physical medium and constitutes part of the channel-coding function.

The data link layer of the OSI model is composed of the following sublayers, ordered from lower to higher: the MAC sublayer, the MAC sublayer, and the Logical Link Control (LLC) sublayer. In some systems, a Reconciliation Sublayer (RS) may also be present to realize the logical connection between the physical layer and the MAC layer; however, this is not a standard component of the OSI model. The MAC sublayer is responsible for controlling access to the physical medium and ensuring correct transmission of data over that medium. The MAC sublayer enables multipoint control and its principal functions include dynamic discovery and registration of ONUs. The LLC sublayer, located above the MAC layer, provides logical link control including frame synchronization, flow control, error control, and bridging as part of adaptation to the network layer.

A core challenge in OFDM-PON is that the maximum achievable data rate for an ONU varies with its distance from the OLT and its specific channel conditions [18]. To manage this complexity in a practical hardware implementation, we introduce a rate-stage model that discretizes the continuum of possible rates into a finite set of operational levels. For this work, we defined five distinct rate stages, each corresponding to a specific combination of subcarrier modulation formats and a resulting theoretical transmission rate, as detailed in Table 1 and Table 2.

### 2.2. Flexible Frame Structure

To support multiple ONUs operating at different rate stages, we designed a novel three-layer header structure for the downstream frame, which has a fixed duration of 31.25 µs.

#### 2.2.1. Downstream Frame

**Figure 2:**
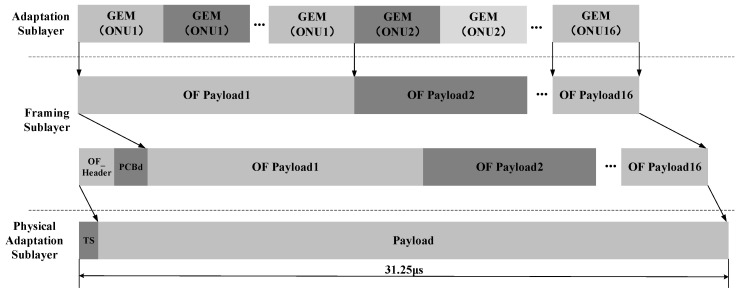
Proposed Downstream Frame Structure.

**Figure 3:**
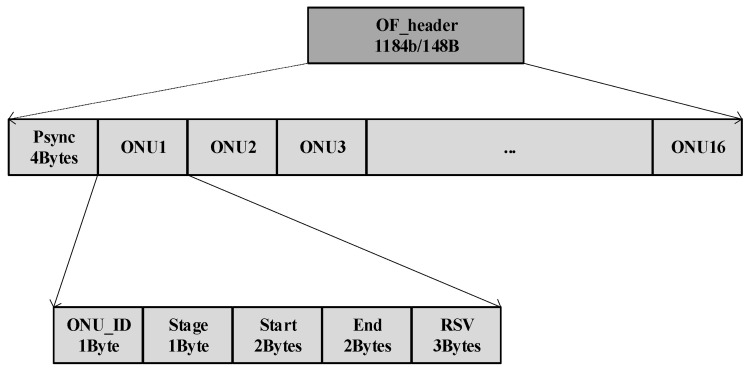
The definition of the OF_header.

As depicted in Figure 2, the downstream frame begins with a Training Sequence (TS) and an OFDM Header (OF_Header). The OF_Header is the key innovation for multi-rate management. The detailed definition of OF_header is explained in Figure 3 where the length of the OF_header is 148 Bytes. The Psync field is used for physical layer synchronization of downlink frames. It consists of 4 Bytes with a fixed value of 0x12345678. The OF_header contains entries for each ONU scheduled in the frame’s payload, specifying the ONU’s ID, its assigned Stage (rate stage), and the Start and End positions of its data block within the payload. This header is always transmitted at the base rate (Stage 0) to ensure all ONUs can decode it.

**Figure 4:**
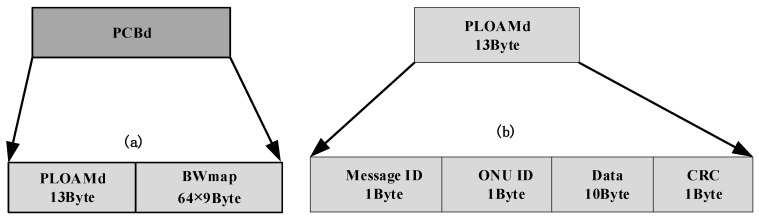
The definition of the PCBd and PLOAMd field. (**a**) PCBd; (**b**) PLOAMd.

Following the header is the Physical Control Block downstream (PCBd), which carries the bandwidth allocation maps (BWmap) and Physical Layer OAM (PLOAMd) messages for system control, similar to GPON. Similar to the OF_Header, PCBd field is also transmitted at the base rate (Stage 0). Shown in Figure 4, the PLOAMd field is a 13-byte block that carries OAM messages between the OLT and each ONU and plays a key role during the system start-up for ONU registration operation. In this field, Message ID field is used to identify the message type and Data field contains the actual control or management information. The detailed definition of the message ID field is shown in Table 3, and the system activation and channel probing procedure based on this field is described in Section 2.3. The final CRC field is used to verify the integrity and correctness of PLOAMd field with the generator polynomial $g\left(x\right)=x^{8}+x^{2}+x+1$.

**Figure 5:**
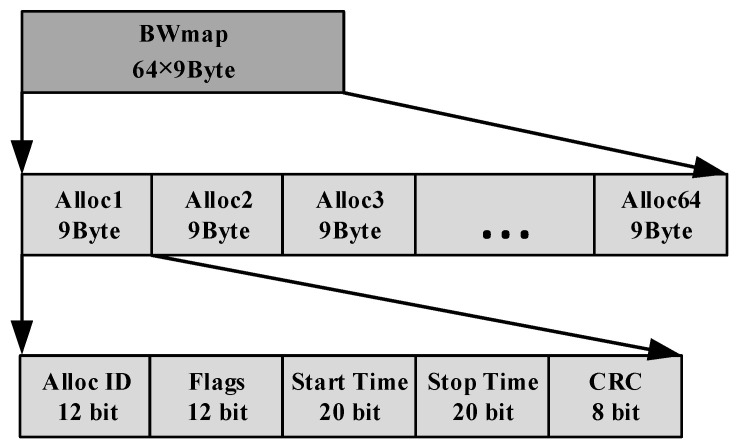
The definition of the BWmap field.

Shown in Figure 5, the BWmap is an array of 64 allocation structures which is used for ONU bandwidth allocation and ONU registration operation. The Alloc-ID field is 12 bits long and used to identify the ONU_ID where the Alloc-ID with 254 is reserved for ONU registration. The Flags field occupies 12 bits which carries the indicator messages needed during ONU activation/registration where bit6 is used for BL_Detecting_Request message, bit5 is used for SN_Request message and bit4 is used for Ranging_Request message. The StartTime and StopTime fields occupy 20 bits counted in user-defined system clock ticks where the StartTime field defines the exact instant the upstream burst begins.

The OF_Payload contains the aggregated GEM frames for multiple ONUs, with each data block transmitted at its designated rate stage shown in Table 1.

#### 2.2.2. Upstream Frame

**Figure 6:**
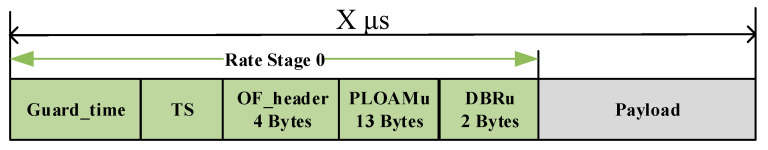
Proposed Upstream Frame Structure.

The upstream channel is divided into time slots allocated by the OLT via the BWmap. Each upstream burst from an ONU consists of a guard time, a training sequence, its own OF_Header, PLOAMu and the data payload. All control information is transmitted at the base rate (Stage 0) to ensure reliable reception by the OLT, while the payload is transmitted at the ONU’s assigned rate stage (see Figure 6).

**Figure 7:**
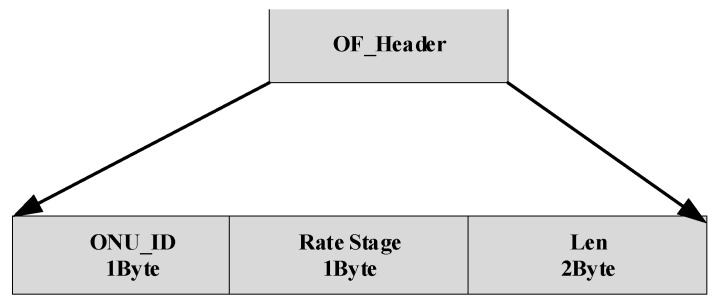
The definition of the OF_header for uplink transmission.

Different with the downlink transmission, guard time is introduced in uplink stream for preventing data collision between consecutive upstream bursts and simultaneously providing the laser-on settling time for the ONU’s transmitter. The OF_Header consists of the ONU_ID field, rate stage field and Len field, shown in Figure 7, where Len indicates the length of the payload for the uplink. The definition of PLOAMu is basically the same as that of PLOAMd, except that the MESSAGE ID message type is different which is shown in Table 4. The DBRu field has a length of 2 Bytes and is used by the ONU to inform the OLT about the amount of buffer space it has available.

### 2.3. System Activation and Channel Probing

**Figure 8:**
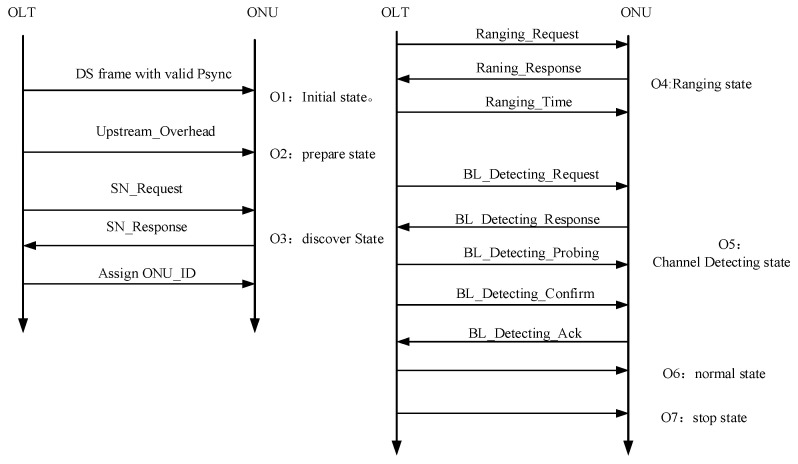
The overall activation process flow for OFDM-PON.

Shown in Figure 8, the ONU activation process follows a state machine progressing from initial discovery to normal operation (O1–O7). Similar to GPON, when the ONU is powered on, it is in the O1 initial state. Once it receives two consecutive frames of downstream data with the correct Psync shown in Figure 3, it will enter the O2 preparation state. In the O2 state, the OLT transmits three consecutive PLOAMd messages to the ONU. These messages are broadcast with the ONU_ID set to 255 and are used to configure pre-assigned delay values for OFDM-PON. Upon receiving at least one valid PLOAM message that passes CRC verification, the ONU transitions to the O3 state. During the O3 state, the OLT initiates a quiet window through the BWmap field to assign serial numbers to the ONUs, after which each ONU transitions to the O4 ranging state. Similarly, a quiet window is required in the O4 state, during which the OLT transmits a ranging request to the ONU. Upon receiving the ONU’s response, the OLT calculates and allocates an appropriate compensation delay. The ONU then proceeds to the O5 channel detecting state.

A novel and essential part of our protocol is the Channel Probing process, which occurs in the O5 state after ranging is complete. This mechanism dynamically determines the highest reliable rate stage for each ONU. The process is managed through a series of newly defined PLOAM messages:

1. The OLT initiates the process by sending a BL_Detecting_Request flag in the BWmap.
2. The OLT then sends a series of BL_Detecting_Probing messages, which have special test frames, to the ONU. It starts with Stage 0 and sequentially increases the rate stage.
3. After receiving each probing frame, the ONU calculates the bit error rate. It then sends a BL_Detecting_Response message back to the OLT, indicating whether the test passed and if it is ready to test the next higher rate.
4. This interactive process continues until the ONU reports a failure (error rate exceeds a threshold) or it has successfully passed the test for the highest rate stage.
5. Finally, the ONU reports the highest successful rate stage to the OLT using a BL_Detecting_Ack message, and the OLT stores this information for future data transmissions.

## 3. FPGA Implementation and Verification

**Figure 9:**
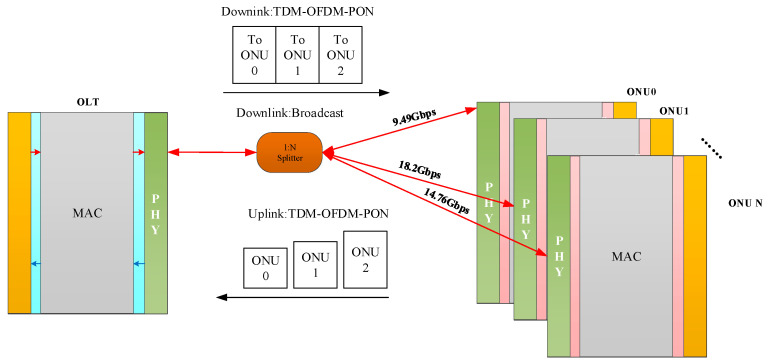
High-level architecture for rate-adaptive OFDM-PON.

Figure 9 illustrates the high-level architecture of the proposed rate-adaptive OFDM-PON system. The network utilizes a Point-to-Multipoint (P2MP) tree topology, where a central Optical Line Terminal (OLT) connects to multiple Optical Network Units (ONUs) through a passive Optical Distribution Network (ODN) consisting of a 1:N optical splitter and fiber links. The system employs a TDM-OFDM-PON transmission scheme for both downstream and upstream links to efficiently manage bandwidth and multiple access.

To validate the proposed protocol, the OLT-side MAC logic was fully implemented in hardware and tested on a Xilinx FPGA development board. The system operates with a core clock frequency of 218.6 MHz. For testing, the OLT MAC’s buffer interface was looped back to an ONU MAC implementation on the same board, emulating a complete PON link. The OLT architecture is divided into downstream and upstream data paths.

- **Downstream Path**: Ethernet traffic, scaled to an equivalent of 40 Gbps, enters the system and is encapsulated into GEM frames. A key component of the implementation is a custom-designed Ring Buffer Pool. This module uses a large on-chip RAM, logically partitioned into smaller blocks, to efficiently buffer incoming data for up to 254 ONUs. It maintains a state table for each ONU, tracking data volume and read/write pointers, allowing for low-latency, non-blocking storage of multi-user traffic. A scheduler reads data from the buffer, which is then processed by the Dynamic Rate Adaptation Module. This module implements the rate-staging by slicing the data and inserting null bits to match the effective throughput required for each specific rate stage before mapping to the physical layer buffer interface.
- **Upstream Path**: The upstream path performs the reverse operations. Data received from the buffer interface is first passed to a Frame Recovery Module. This critical module parses the rate-stage information from the burst header and performs precise bit-shifting and masking operations to discard the null bits and perfectly reconstruct the original, contiguous GEM frames. The recovered GEM frames are then converted back to Ethernet frames and sent to the network interface.
- **Activation Management Module**: A finite state machine implemented in hardware controls the entire ONU activation process, including the sequencing of PLOAM messages for ranging and the interactive channel sounding procedure.

The final implementation was synthesized and placed on the FPGA, with resource utilization shown in Table 5. The design makes significant use of Block RAM for the buffer pool but is otherwise efficient in its use of logic resources.

## 4. Experimental Results and Discussion

The implemented system was tested using a Xena Networks TestStorm network analyzer.

### 4.1. Functional Verification

**Figure 10:**
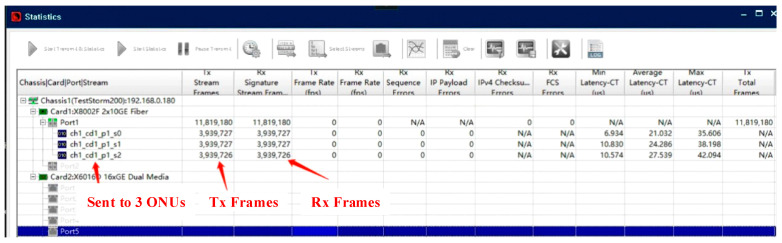
Downstream Link Correctness Test Result.

**Figure 11:**
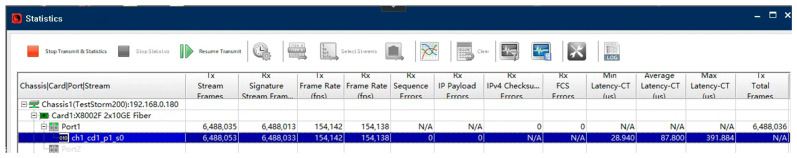
Upstream Link Correctness Test Result.

We first verified the protocol’s logical correctness using a full loopback test. The network analyzer transmitted Ethernet frames with random lengths (64 to 1518 Bytes) to the OLT MAC, which then scheduled them for three different ONUs. The data was looped back from the ONU MACs to the OLT and finally returned to the analyzer. As shown in Figure 10 and Figure 11, the total number of transmitted frames perfectly matched the sum of received frames for each ONU, with zero packet loss, confirming the correctness of the data path for both upstream and downstream links.

### 4.2. Performance Evaluation

**Figure 12:**
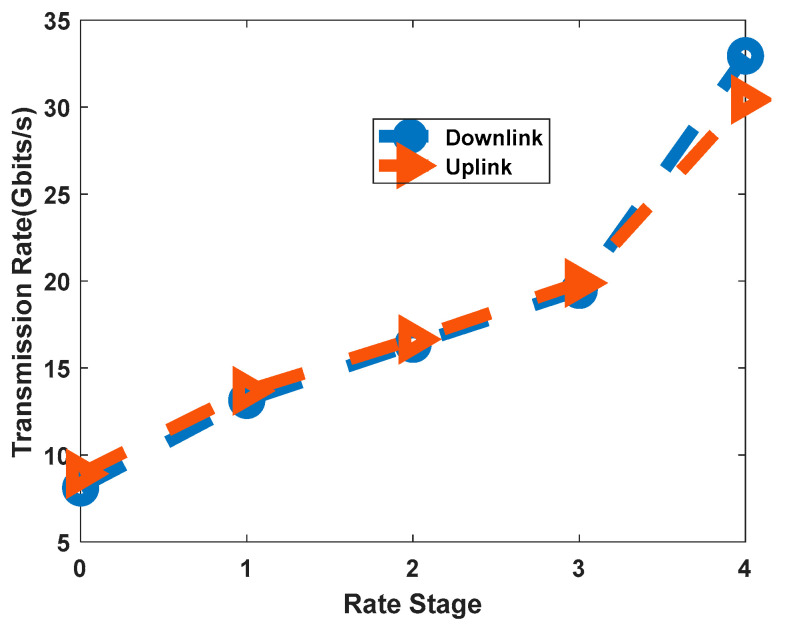
Measured Throughput for Random Frame Lengths.

Next, we measured the maximum achievable net data rate for each of the five rate stages. The network analyzer sent traffic at an increasing rate until packet loss was detected. Figure 12 plots the maximum error-free throughput for both uplink and downlink with random frame lengths. The results demonstrate the system’s ability to achieve flexible rate transmission. The measured downlink throughput scaled from 8.1 Gbit/s at Stage 0 to a maximum of 32.8 Gbit/s at Stage 4. The slight deviation from the theoretical values in Table 1 is attributed to the overhead of the fixed-rate headers and control fields within the frame structure.

**Figure 13:**
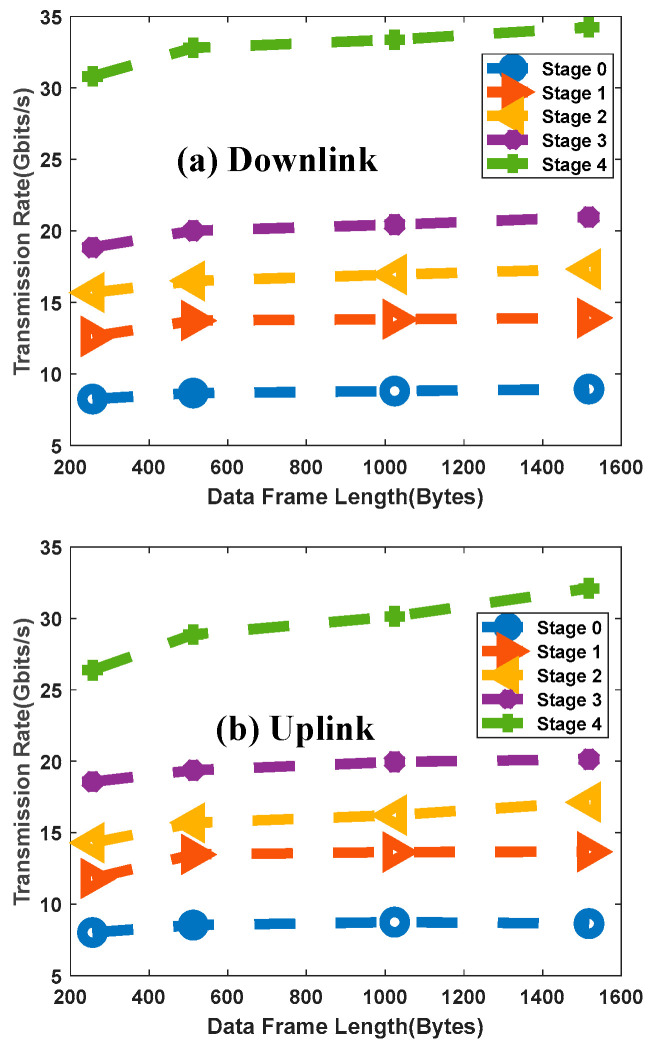
Measured Throughput for Fixed Frame Lengths.

We also investigated the impact of Ethernet frame size on performance, with results for the uplink/downlink shown in Figure 13. For any given rate stage, throughput increases with frame size and then plateaus. This is a typical behavior in hardware-based packet processing systems, where the fixed per-packet processing overhead becomes less significant for larger packets, leading to higher efficiency. The tests confirm that the system can sustain high throughput across various traffic profiles.

## 5. Conclusions

This paper has presented the design, hardware implementation, and experimental validation of a novel rate-adaptive MAC protocol for flexible OFDM-PON systems. By introducing a physical adaptation sublayer, a discretized rate-stage model, and a flexible three-layer frame structure, our protocol effectively manages the variable, distance-dependent data rates that are characteristic of OFDM-PONs. A key innovation is the dynamic channel sounding mechanism, which allows the system to determine and assign the optimal transmission rate for each ONU during activation.

The protocol was successfully implemented on an FPGA platform and verified through comprehensive hardware testing. The results confirm the protocol’s correctness and demonstrate its ability to achieve flexible downlink data rates ranging from 8.1 Gbit/s to 32.8 Gbit/s. This work provides a complete and validated solution that bridges a critical gap between physical layer capabilities and the practical system-level requirements for deploying next-generation optical access networks. Future work will focus on incorporating more advanced dynamic bandwidth allocation (DBA) algorithms and integrating this MAC layer with a real-time physical layer transceiver.

## Figures and Tables

**Table 1:** Modulation formats and bit loading for subcarrier.

Rate Stage	BPSK /Subcarrier	QPSK /Subcarrier	8QAM /Subcarrier	16QAM /Subcarrier
0	304	0	0	0
1	473	169	0	0
2	583	59	110	0
3	700	61	100	45
4	1144	10	10	270

**Table 2:** Rate Stage to Theoretical Throughput Mapping.

Rate Stage	Effective Bits	Theoretical Transmission Rate
0	304	9.49 Gbps
1	473	14.76 Gbps
2	583	18.20 Gbps
3	700	21.85 Gbps
4	1144	35.71 Gbps

**Table 3:** The definition of the message ID for downlink transmission.

Message ID Value	Message Type	Function
1	SN_Request	Quiet window message generated by OLT for ONU to generate the SN_Response Message
2	Assign ONU_ID	The ONU ID allocation message generated by OLT
3	Ranging_Request	Ranging request message generated by OLT for calculation of the Round-trip-delay between the OLT and ONU
4	Ranging_Time	The Round-trip-delay allocation message generated by OLT
5	BL_Detecting_Request	The bit loading request message generated by OLT
6	BL_Detecting_Probing	The special test frames generated by OLT
7	BL_Detecting_Confirm	The bit loading results generated by OLT

**Table 4:** The definition of the message ID for uplink transmission.

Message ID Value	Message Type	Function
1	SN_Response	Response message for the SN_Request message generated by OLT
2	Ranging_Response	Response message for the Ranging_Request message generated by OLT
3	BL_Detecting_Response	Response message for the BL_Detecting_Request message generated by OLT
4	BL_Detecting_Ack	ack message for the BL_Detecting_Confirm message generated by OLT

**Table 5:** FPGA Resource Utilization.

Resource Type	Used	Available	Utilization
LUT	125,232	1,182,240	10.59%
Flip-Flops	167,481	2,364,480	7.08%
Ultra RAM	48	960	5.00%
Block RAM	1464.4	2160	67.80%

## Data Availability

The raw data supporting the conclusions of this article will be madeavailable by the authors on request.

## Abbreviations

The following abbreviations are used in this manuscript:

OFDM	Orthogonal Frequency Division Multiplexing
PON	Passive Optical Network
MAC	Medium Access Control
ONU	Optical Network Unit
OLT	Optical Line Terminal
TDM	Time Division Multiplexing
GEM	GPON Encapsulation Method
OF_Header	OFDM Header
PCBd	Physical Control Block downstream
OAM	Operations, Administration and Maintenance
PLOAMd	Physical Layer Operations, Administration and Maintenance downstream
BWmap	Bandwidth Map

## References

[1] Liu G., Wang G., Huang Y., He J., Bo Y., Zheng K., Li M., Wu Y., Lu Y., Ye Z. World’s First Demonstration of Real-time Symmetric Flexible Rate PON with Entropy-Loading and 10G-class Optics. Proceedings of the 2022 European Conference on Optical Communication (ECOC).

[2] Lee J., Park J., Han S.-K. (2025). OFDM-NOMA Optical Transmission Utilizing Dispersion-Induced Power Fading of SMF for Multi-Distance PON. J. Light. Technol..

[3] Hajduczenia M., da Silva H.J.A. Next generation PON systems—Current status. Proceedings of the 2009 11th International Conference on Transparent Optical Networks.

[4] Zhu M., Gu J., Chen B., Gu P. (2022). Dynamic subcarrier assignment in OFDMA-PONs based on deep reinforcement learning. IEEE Photonics J..

[5] Breuer D., Geilhardt F., Hulsermann R., Kind M., Lange C., Monath T., Weis E. (2011). Opportunities for next-generation optical access. IEEE Commun. Mag..

[6] Weis E., Breuer D., Lange C. Technologies for next generation optical access. Proceedings of the 2012 14th International Conference on Transparent Optical Networks (ICTON).

[7] Zhou J., He J., Lu X., Wang G., Bo Y., Liu G., Huang Y., Li L., Yang C., Wang H. (2022). 100G fine-granularity flexible-rate passive optical networks based on discrete multi-tone with PAPR optimization. J. Opt. Commun. Netw..

[8] Jin X.Q., Tang J.M. (2012). Experimental investigations of wavelength spacing and colorlessness of RSOA-based ONUs in real-time optical OFDMA PONs. J. Light. Technol..

[9] Zhang J., Yi X., Yang Q., Zhang H., Deng M., Qiu K. (2012). Collective reception of orthogonal band multiplexed data streams for OFDM-PON upstream transmissions. Opt. Commun..

[10] Chen L., Qiao Y., Zhao Y., Ji Y. (2012). Wide-range frequency offset estimation method for a DD-OFDM-PON downstream system. J. Opt. Commun. Netw..

[11] Chen M., He J., Tang J., Wu X., Chen L. (2014). Experimental demonstration of real-time adaptively modulated DDO-OFDM systems with a high spectral efficiency up to 5.76 bit/s/Hz transmission over SMF links. Opt. Express.

[12] Di X., Chen L., Xiao J., Chen M., He J., Yu J., Cheng Y. (2013). A novel timing offset estimation method for direct-detection optical OFDM systems. Opt. Fiber Technol..

[13] Chanclou P., Cui A., Geilhardt F., Nakamura H., Nesset D. (2012). Network operator requirements for the next generation of optical access networks. IEEE Netw..

[14] Zhang J., Wang T., Ansari N. An efficient MAC protocol for asynchronous ONUs in OFDMA PONs. Proceedings of the 2011 Optical Fiber Communication Conference and Exposition and the National Fiber Optic Engineers Conference.

[15] Kanonakis K., Cvijetic N., Tomkos I., Wang T. (2013). Dynamic Software-Defined Resource Optimization in Next-Generation Optical Access Enabled by OFDMA-Based Meta-MAC Provisioning. J. Light. Technol..

[16] Nunes R.B., Bacalhau J.M., Silva J.A., Segatto M.E. (2017). A MAC layer protocol for a bandwidth scalable OFDMA PON architecture. Comput. Commun..

[17] Lim W., Shin S., Yang Y.-M. MAC protocol designs for OFDMA-PONs. Proceedings of the 2014 14th International Symposium on Communications and Information Technologies (ISCIT).

[18] Cui H., Zhou J., Mo W., Qiao Y. (2024). Flexible-Rate PON Based on Entropy-Loading DMT with Wide-Range Adjustment From 12.5 to 87.5 Gbps. IEEE Photonics Technol. Lett..

